# Characterization of atypical T cells generated during *ex vivo* expansion process for T cell-based adoptive immunotherapy

**DOI:** 10.3389/fimmu.2024.1202017

**Published:** 2024-03-13

**Authors:** Patricia Mercier-Letondal, Abhishek Kumar, Chrystel Marton, Francis Bonnefoy, Maxime Fredon, Laura Boullerot, Barbara Dehecq, Olivier Adotévi, Yann Godet, Jeanne Galaine

**Affiliations:** ^1^ UMR 1098 RIGHT INSERM, Etablissement Français du Sang Bourgogne Franche-Comté, Université de Franche-Comté, Besançon, France; ^2^ Centre Hospitalier Universitaire de Lille, Service des Maladies du Sang, Lille, France; ^3^ Centre Hospitalier Universitaire de Besançon, Service D’oncologie Médicale, Besançon, France

**Keywords:** T cell-based adoptive cell immunotherapy, transgenic TCR T cells, CD8 and CD4 expression, MHC restriction, *ex vivo* T cell culture process, T cell function

## Abstract

Engineered T cell-based adoptive immunotherapies met promising success for the treatment of hematological malignancies. Nevertheless, major hurdles remain to be overcome regarding the management of relapses and the translation to solid tumor settings. Properties of T cell-based final product should be appropriately controlled to fine-tune the analysis of clinical trial results, to draw relevant conclusions, and finally to improve the efficacy of these immunotherapies. For this purpose, we addressed the existence of atypical T cell subsets and deciphered their phenotypic and functional features in an HPV16-E7 specific and MHC II-restricted transgenic-TCR-engineered T cell setting. To note, atypical T cell subsets include mismatched MHC/co-receptor CD8 or CD4 and miscommitted CD8+ or CD4+ T cells. We generated both mismatched and appropriately matched MHC II-restricted transgenic TCR on CD8 and CD4-expressing T cells, respectively. We established that CD4+ cultured T cells exhibited miscommitted phenotypic cytotoxic pattern and that both interleukin (IL)-2 or IL-7/IL-15 supplementation allowed for the development of this cytotoxic phenotype. Both CD4+ and CD8+ T cell subsets, transduced with HPV16-E7 specific transgenic TCR, demonstrated cytotoxic features after exposure to HPV-16 E7-derived antigen. Ultimately, the presence of such atypical T cells, either mismatched MHC II-restricted TCR/CD8+ T cells or cytotoxic CD4+ T cells, is likely to influence the fate of patient-infused T cell product and would need further investigation.

## Introduction

For the past decade, numerous breakthroughs in cancer therapy have occurred. Among them, the area of T cell-based Adoptive Cell Therapy (ACT) has particularly met promising success. Tumor-associated antigen (TAA) specific ACT includes *ex vivo* expanded Tumor Infiltrating Lymphocytes (TIL), or engineered-T cells such as Chimeric Antibody Receptor (CAR-T) and transgenic T Cell Receptor expressing T cells (TCR-T), both reprogrammed to target TAA (1).

TCR-T cells are engineered to express a TCR derived from a TAA-specific T cell clone (2) to target extra or intracellular antigens, in an MHC-dependent manner. The first encouraging clinical trial of TCR-T infusion was performed in 2008 in metastatic melanoma (3). To date, many clinical trials are ongoing in the field of TCR-T-based ACT (2, 4, 5) and some of them have already displayed promising clinical results in the case of, for instance, MAGE-A3 or NY-ESO-1 expressing cancers (6, 7) or high-risk Human Papillomavirus (HPV) related malignancies (8).

TCR-T are usually isolated from a T cell clone, either MHC I-restricted (TCR I) CD8+ T cells or MHC II-restricted (TCR II) CD4+ T cells, depending on whether a cytotoxic or a helper function is initially expected. The canonical role of CD8+ and CD4+ T cells is to eliminate pathogenic cells *via* cytotoxic mechanisms and to coordinate a specific immune global response, respectively. However, these roles can be blurred for non-conventional reprogrammed T cells. On the one hand, the TCR-T vector can integrate the CD8+ or CD4+ T cell genome, regardless of its MHC class I or II restrictions. Consequently, mismatched TCR-T I/CD4+ and TCR-T II/CD8+ engineered T cells can be generated at an expected similar level to matched TCR-T I/CD8+ or TCR-T II/CD4+ T cells. This MHC/co-receptor mismatch is likely to impact the functions of engineered TCR-T cells. On the other hand, standard IL-2 supplementation of culture medium during the stage of *ex vivo* expansion of engineered T cells is prone to induce cytotoxic (CTX) CD4+ T cells (9, 10).

Previously, atypical T cells, namely T cells that do not behave as usually expected according to matching and commitment-associated rules, have already been described in several physiological and pathological settings. Thus, regarding CD4+ and CD8+ T cells, it has been documented that naturally mismatched TCR-T I/CD4+ and TCR-T II/CD8+ can occur and both of them behave mainly like classical CTX CD8+ T cells (11–15). This mismatched T cell generation has also previously been reported in transgenic contexts, along with CD8+ T cell-associated characteristics (16–24). Moreover, irrespective of defined canonical functions, miscommitted T cells (*i.e.* T cells exhibiting a different role than the canonical one) have already been described. Indeed, natural CD8+ T cells displaying helper features have been identified (25). Similarly, it has been reported more than 30 years ago that CD4+ T cells are able to mount an antigen-specific cytotoxic response in diverse infectious settings, as reviewed by Juno and colleagues (26). This characterization has been recently confirmed in the context of cancer antigen recognition (27). Oh & Fong (10) reviewed current knowledge on the topic and described a cytotoxic-associated CD4+ T cell phenotypic pattern.

Our team previously developed (17) an MHC II-restricted HPV-16 E7 TCR-T and demonstrated both CD8+ and CD4+ engineered-T cell specificity and functionality in terms of cytokine secretion after co-culture with relevant antigen-bearing target cells. Here, we aim to unravel cytotoxic features of mismatched TCR II/CD8+ along with matched TCR II/CD4+ transduced T cells, and thereafter focus on CD4 T cells to assess the role of *ex vivo* production process on CD4+ T cell cytotoxic polarization.

## Materials and methods

### Biological material

Peripheral blood mononuclear cells (PBMC) were collected from healthy donors at the Etablissement Français du Sang (EFS) as apheresis kit preparations after informed consent and according to the collection agreement AC-2020-4129. EBV-transformed B lymphoblastoid cell line (BLCL) was generated from an HLA-DRB1*04 healthy donor PBMC as previously described (28). The SKMEL-28 cell line, known to express HLA-DRB1*04 at its surface, was obtained from ATCC (HTB-72) and cultured according to manufacturer’s instructions. Both cell lines were periodically checked for mycoplasma contamination. NOD/SCID IL-2Rγ^-/-^ (NSG) mice were bred in the animal facility of the University of Franche-Comté, according to the approved experimental project 2021‐004‐OA12PR.

### Peptides

HPV16-E7_70-89_ peptide (QSTHVDIRTLEDLLMGTLGI) was selected as previously described (17) and purchased from Proteogenix. Peptide purity is superior to 90%.

### Retroviral vector

TCR α and β chains obtained from HPV16-E7-specific and HLA-DRB1*04-restricted CD4 T cell clones were introduced into a pSFG retroviral vector backbone, along with ΔCD19 selection and tracking marker, as previously described (17).

HPV-16 E7 encoding pLXSN plasmid was kindly supplied by Dr. A. Baguet (UMR RIGHT). The Neomycin resistance (NeoR) gene is included in the vector, as a selection gene.

### T cell activation, retroviral transduction, selection, and expansion

Healthy donor T cells were magnetically isolated and activated by using CD3/CD28 microbeads (Fisher Scientific, 111.31D) according to manufacturer’s instructions. Beads-attached T cells were cultured in RPMI-1640 medium (Fisher Scientific, 11544526) with 10% human serum (local production) in presence of 500 IU/mL Interleukin (IL)-2 (Clinigen Healthcare BV, Proleukin^®^) or 350 IU/mL IL-7 (Miltenyi Biotec, 130-095-362) + 60 IU/mL IL-15 (Miltenyi Biotec, 130-095-762), according to specific experiment. The complete medium was renewed every 2-3 days until day 10 of culture. At day 2, activated and IL-2-cultured cells were transduced (GMTC) using HPV16-E7/HLA-DRB1*04-specific TCR retroviral supernatant, whose retroviral particles were trapped on RetroNectin^®^ (Takara, T100B). At day 6, transduction efficiency was assessed through membrane staining with CD3 BV421 (BD Biosciences, 562426), and CD19 APC (Miltenyi Biotec, 130-113-165) antibodies, and analyzed by flow cytometry (FCM). Transduced T cells were then magnetically sorted using CD19 microbeads (Miltenyi Biotec, 130-050-301) following manufacturer’s instructions. Sorting efficiency was performed through the same FCM analysis as transduction efficiency. At day 10, T cell bulk composition was evaluated *via* membrane staining with CD3 BV421, CD4 FITC (Diaclone, 954.031.010) and CD8 PE (Diaclone, 854.962.010) antibodies. An activated and untransduced cellular counterpart (UTC) from the same donor was also cultured for all experiments as a negative control of the anti-tumoral effect.

### CD4+ and CD8+ T cells sorting

T cells were stained with CD4 FITC and CD8 PE antibodies according to manufacturer’s instructions and resuspended in PBS 1X (Fisher Scientific, 11530546) 2mM EDTA. CD8+/CD4- and CD8-/CD4+ cells were further sorted using an FCM-based cell sorter (Sony, SH800) and cultured for four additional days in complete medium with 500 IU/mL IL-2. A sorted cell sample was set aside to assess enrichment efficacy through FCM analysis.

### Phenotypic and functional assessment of cultured and engineered- T cells

Regarding IL-2 cultured TCR-T or UTC, the exhaustion-associated phenotype was evaluated through a staining with Fixable viability Dye (FvD) eFluor780 (Life Technologies, 65-0865-14), and CD3 FITC (BD Biosciences, 555332), CD8 BV510 (BD Biosciences, 563919), CD19 APC, anti-PD-1 PE-Cy7 (BD Biosciences, 561272), anti-TIM-3 PerCP-Cy5.5 (Sony, RT2325080), anti-TIGIT BV421 (BD Biosciences, 747844) antibodies and analyzed by FCM. CD8+ and CD4+ TCR-T cell exhaustion score was calculated as described by Chen et al. (29). Briefly, the formula is (where MFI is the mean fluorescence intensity):


$$\text{Exhaustion score}=\left({\text{MFI}}^{\text{PD-}1}/{\text{MFI}}^{\text{CD}19}+{\text{MFI}}^{\text{TIM-}3}/{\text{MFI}}^{\text{CD}19}+{\text{MFI}}^{\text{TIGIT}}/{\text{MFI}}^{\text{CD}19}\right)/3$$

The activation-related phenotype was assessed through a staining with FvD eFluor780, CD19 APC, CD3 BV421, CD8 BV510, CD25 FITC (Sony, RT2113020), CD69 APC-R700 (BD Biosciences, 565154) and anti-HLA-DR PE (BD Biosciences, 555561) antibodies before FCM analysis. CD4+ and CD8+ TCR-T cell activation score was obtained through the formula given by Chen et al. (29). Briefly, the formula is:


$$\text{Activation score}=\left({\text{MFI}}^{\text{CD}25}/{\text{MFI}}^{\text{CD}19}+{\text{MFI}}^{\text{CD}69}/{\text{MFI}}^{\text{CD}19}+{\text{MFI}}^{\text{HLA DR}}/{\text{MFI}}^{\text{CD}19}\right)/3$$

Transgenic TCR functionality was evaluated by co-culturing IL-2-exposed sorted CD4+ and CD8+ T cells, both transduced and untransduced, with HPV16-E7_70-89_ peptide-pulsed (2µM) or unpulsed allogeneic HLA-DRB1*04 BLCL, at the effector:target (E:T) ratio of 1:1, as previously described (17). CD107a expression was assessed by adding Golgi Stop (BD Biosciences, 554724) and CD107a PE antibody (BD biosciences, 555801) simultaneously to cell culture during a 5-hour co-culture before staining with FvD eFluor780, CD3 BV421 and CD19 APC antibodies prior to FCM analysis. Cytotoxicity assay was performed after an overnight co-culture through CD3 BV421 and CD19 APC antibodies, Annexin-V FITC, and 7-AAD (Beckman Coulter, IM3614) cell staining. Additionally, peptide-pulsed or not and CFSE-stained target cell lysis was evaluated by a Trucount™ device (BD Bioscience, 340334) after an overnight co-culture with TCR-T cells or UTC (E:T ratio from 1:1 to 1:5).

The phenotype and functionality of resting PBMC-derived T cells and IL-2 or IL-7+IL-15 cultured UTC cells were assessed after a 5-hour stimulation with 25ng/mL PMA (Sigma-Aldrich, P8139) and 1.25µg/mL ionomycin (Sigma Aldrich, I0634) and treatment with Golgi Stop. CD107a expression was assessed after 5 hours through staining with CD107a PE-CF594 antibody (BD Biosciences, 562628) according to manufacturer instructions, before staining with FvD eFluor780, CD3 BV421, and CD4 FITC antibodies. Cytotoxicity-associated CD4+ T cell phenotype was evaluated after staining with FvD, CD3, CD4, CD8, CD137, CD134, anti-TRAIL, anti-FasL, anti-SLAMF7 antibodies, followed by intracellular staining with anti-Granzyme B and anti-Perforin, using a fixation and permeabilization kit (BD Biosciences, 550028); panel 1 and panel 2 are further described in **Table 1**.

**Table 1:** Antibody panels used for cytotoxic-associated CD4+ T cell phenotypic evaluation.

	Panel 1			Panel 2		
	Fluorochrome	Supplier	Reference	Fluorochrome	Supplier	Reference
**FvD**	Alexa Fluor 700	BD Biosciences	564997	eFluor780	Life Technologies	65-0865-14
**CD3**	APC-Cy7	Biolegend	300470	BV421	BD Biosciences	562426
**CD4**	BV510	BD Biosciences	562970	FITC	Diaclone	954.031.010
**CD8**	BV786	Biolegend	344740	ND	ND	ND
**anti-SLAMF7**	PE	Biolegend	331806	PE	Sony	RT2259030
**anti-Granzyme B**	PE-CF594	BD Biosciences	562462	BV510	BD Biosciences	563388
**anti-perforin**	Alexa Fluor 488	BD Biosciences	563764	ND	ND	ND
**CD134 (OX40)**	BV605	Biolegend	350028	ND	ND	ND
**CD137 (4-1BB)**	PE-Cy7	Biolegend	309818	ND	ND	ND
**anti-TRAIL**	BV650	BD Biosciences	743721	ND	ND	ND
**anti-FasL**	BV421	Biolegend	306411	ND	ND	ND

### Target cell line generation and mouse model design for *in vivo* CD4+ and CD8+ TCR-T cells functionality evaluation

SKMEL-28 cell line was checked for HLA-DR expression (HLA-DR PE). Its capacity to present HPV-16 E7_70-89_ to TCR-T cells was assessed after pulsing SKMEL-28 cells with 2µM peptide and a co-culture with Golgi Plug-treated TCR-T cells, as well as UTC (18h, 37°C). IFN-γ secretion was assessed after membrane staining with FvD eFluor780, CD3 BV421, CD4 FITC, and CD8 PE antibodies followed by intracellular staining (BD Biosciences, 550028) with anti-IFN-γ APC antibody (BD Biosciences, 554702), and FCM analysis.

SKMEL-28 cells were transfected with HPV-16 E7 pLXSN encoding DNA plasmid vector using Lipofectamine™ LTX reagent (Thermo Fisher, A12621), selected during 3 weeks with 1mg/mL Geneticin (Thermo Fisher, 10092772) and evaluated for HPV-16 E7 expression through western blotting, as previously described (17).

Two million HPV-16 E7-expressing SKMEL-28 cells were subcutaneously injected into 8-week-old female NSG mice (Charles River, 614NSG) in the presence of Matrigel™ (Thermo Fisher, 11593620). Three to five mice per group were analyzed. After tumors developed in mice flank, 5*10^6^ sorted CD4+ and CD8+, or total unsorted TCR-T or UTC cells were intravenously injected. Ten µg HPV-16 E7_70-89_ were injected at the tumor site 2 hours before T cell infusion to potentiate TCR-T cells-mediated immune response against HPV-16 E7-expressing tumor. A second TCR-T injection was performed 7 days after the first one, regarding all GMTC (*i.e.* IL-2-cultured and transduced cells) fractions and the total UTC control group. Tumor volume was monitored for 17 days according to the following formula before mice sacrifice and TCR-T cell tumor-infiltration evaluation (L and l mean tumor length and width, respectively):


$$\text{Tumor volume}=\text{L}\times {\text{l}}^{2}\times \text{π}/6$$

Upon subsequent mice sacrifice, tumors were harvested and thereby disrupted using a Tumor Dissociation Kit (Miltenyi Biotec, 130-096-730), according to manufacturer instructions. FvD eFluor780, anti-human CD45 BV510 (Sony, RT2120180), anti-mouse CD45 PE-Cy7 (Sony, RT1115570), CD3 BV421, CD4 FITC, CD8 PE and anti-murine constant TCR β chain APC (BD Biosciences, 553174 – transgenic TCR construct contains a murine constant β chain to avoid TCR mispairing between the endogenous and the transgenic TCRs) antibodies were used along with Trucount™ tubes to stain tumor cell extract before FCM analysis.

### Flow cytometry analysis

Appropriate isotypic controls were included in all staining designs.

Staining implying CD107a PE-CF594 antibody, as well as exhaustion, activation panels, and mice-injected TCR-T follow-up, were acquired on a BD FACS LSR Fortessa flow cytometer and analyzed through BD FACS Diva software (version 8.0).

Cytotoxic-associated CD4+T cells phenotypic evaluation panel 1 was assessed through a Beckman Coulter CytoFLEX LX flow Cytometer and analyzed through Kaluza software (version 2.1).

All additional stainings were acquired using a BD FACS CANTO II flow cytometer and analyzed with BD FACS Diva software (version 8.0).

### Statistical analysis

Statistical analysis were performed through Graphpad Prism v9 software and consisted, as specifically mentioned in figure captions, of a one or two-tailed and paired or unpaired t-test. An Aspin Welch correction was applied in case of heterogeneous standard deviation between the compared groups. P-values< 0.05 were considered to be statistically significant. P-values between 0.05 and 0.1 were considered to be testifying to a trend towards statistical significance. P-values > 1 were considered to be statistically non-significant and are not mentioned.

## Results

### Post-*ex vivo* expansion and transduction T cell bulk composition

**Figure 1:**
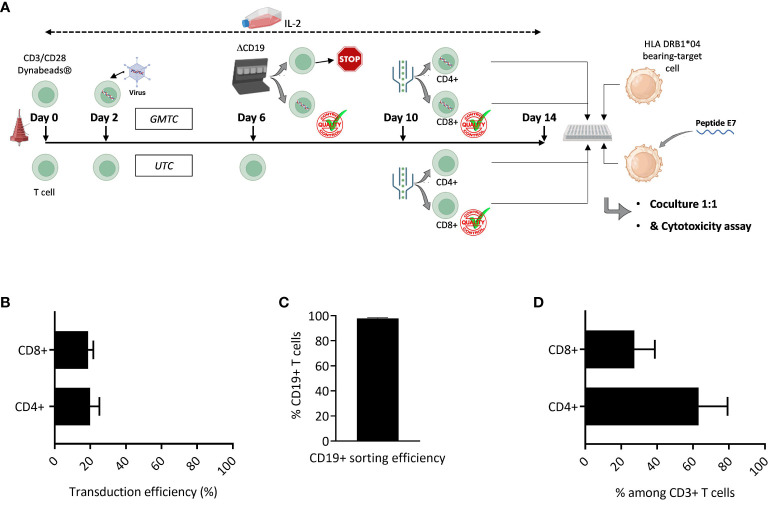
Final T cell bulk characterization. T cells are activated, cultured in the presence of IL-2, transduced with HPV16-E7/HLA-DRB1*04-specific TCR-T and selected on the basis of ΔCD19 expression. **(A)** Graphic representation of experiment design. Figure adapted from images created with BioRender.com. **(B)** CD4+ and CD8+ T cell transduction efficiency is evaluated through the expression of ΔCD19 selection gene among CD3+ CD4+ or CD3+ CD8+ T cells by flow cytometry; data represent mean+/-SD from 3 independent experiments; two-tailed paired t-test. **(C)** ΔCD19-based sorting efficiency; data represent mean+/-SD from 4 independent experiments. **(D)** CD4+ & CD8+ T cell percentage among T cell bulk is evaluated by the expression of CD4 and CD8 co-receptors among CD3+ T cells by flow cytometry; data represent mean+/-SD from 7 independent experiments.

Gene-modified TCR-T along with untransduced control T cells exposed to IL-2 (**Figure 1A**) were evaluated for CD8+ and CD4+ T cell composition. The retroviral vector is likely to transduce both CD4+ and CD8+ T cells in a similar fashion (**Figure 1B**). Subsequent ΔCD19-based GMTC sorting allows for a high purity percentage of GMTC, with a mean of 97.88%+/-0.48 (SD) (**Figure 1C**). The final TCR-T cell product is thus constituted of both CD4+ and CD8+ T cells (**Figure 1D**).

### Phenotypic characterization of CD4+ and CD8+ TCR-T cells

**Figure 2:**
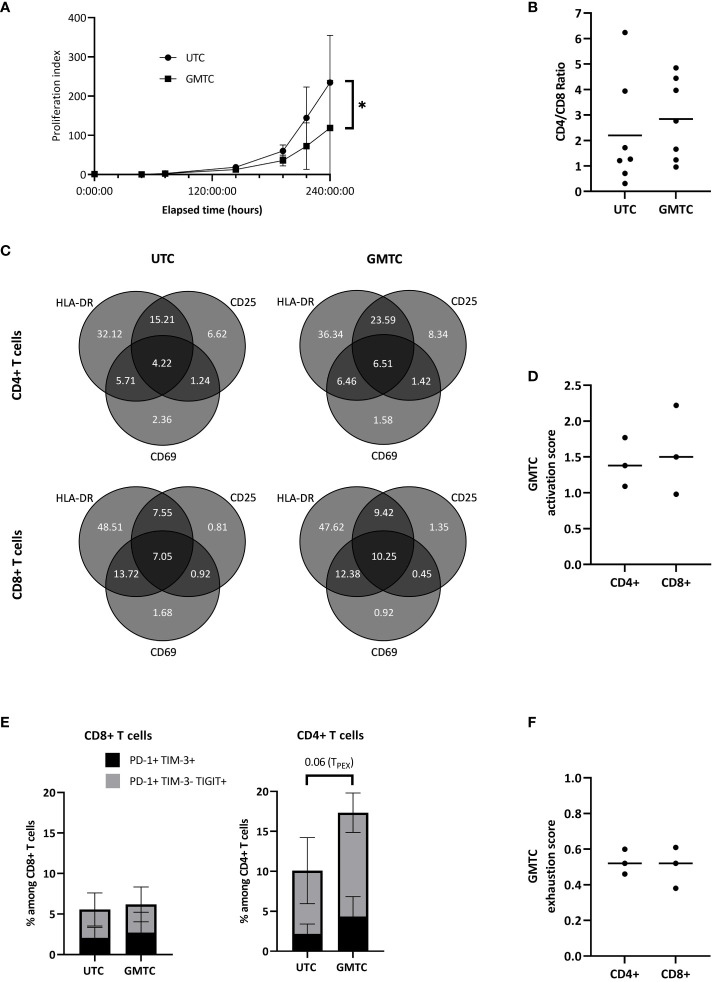
Phenotypic characterization of transduced CD8+ and CD4+ T cells. T cells are activated, cultured in the presence of IL-2, and transduced with HPV16-E7/HLA-DRB1*04-specific TCR-T. **(A)** Selected transduced (GMTC) and untransduced (UTC) T cells culture-related fold expansion; data represent mean+/-SD from 3 independent experiments; two-tailed paired t-test, *: p< 0.05. **(B)** Selected transduced and untransduced T cell CD4/CD8 ratio. Data represent individual values and mean from 7 independent experiments; two-tailed paired t-test. **(C)** Transduced and untransduced, CD4+ and CD8+, T cell activation pattern expression; data represent mean from 3 independent experiments; two-tailed paired t-tests for each subset. **(D)** Activation score of CD4+ and CD8+ TCR-T cells; data represents individual values and median from 3 independent experiments; two-tailed paired t-test. **(E)** Transduced and untransduced, CD4+ and CD8+, T cell exhaustion pattern expression; data represent mean+/-SD from 3 independent experiments; two-tailed paired t-test. **(F)** Exhaustion score of CD4+ and CD8+ TCR-T cells; data represents individual values and median from 3 independent experiments; two-tailed paired t-test.

First, GMTC expansion capacities are lower than those of UTC (p = 0.011) (**Figure 2A**). GMTC and UTC CD4/CD8 ratios are 2.84 [0.96-4.85] and 2.20 [0.31-6.24], respectively (**Figure 2B**); this difference is not statistically significant. The phenotypic profile of activation evaluation does not show any differences between CD4+ and CD8+ T cells, either transduced or not, regarding CD25, CD69, and HLA-DR expression patterns (**Figure 2C**). Moreover, CD4+ and CD8+ GMTC activation scores are not statistically different (**Figure 2D**). CD8+ GMTC and UTC exhaustion phenotypes are similar in terms of PD-1+ TIM-3+ cell population (exhausted T cells, T_EX_) or PD1+ TIM-3- TIGIT+ cell population (progenitor exhausted T cells, T_PEX_). A trend to increase is revealed regarding CD4+ TCR-T_PEX_ compared to CD4+ UTC-T_PEX_ (p=0.06), but not CD4+ TCR-T_EX_ (**Figure 2E**). Exhaustion scores are similar for CD4+ and CD8+ TCR-T cells (**Figure 2F**).

### *In vitro* cytotoxic capacity characterization of both CD8+ and CD4+ TCR-T II transduced T cells

**Figure 3:**
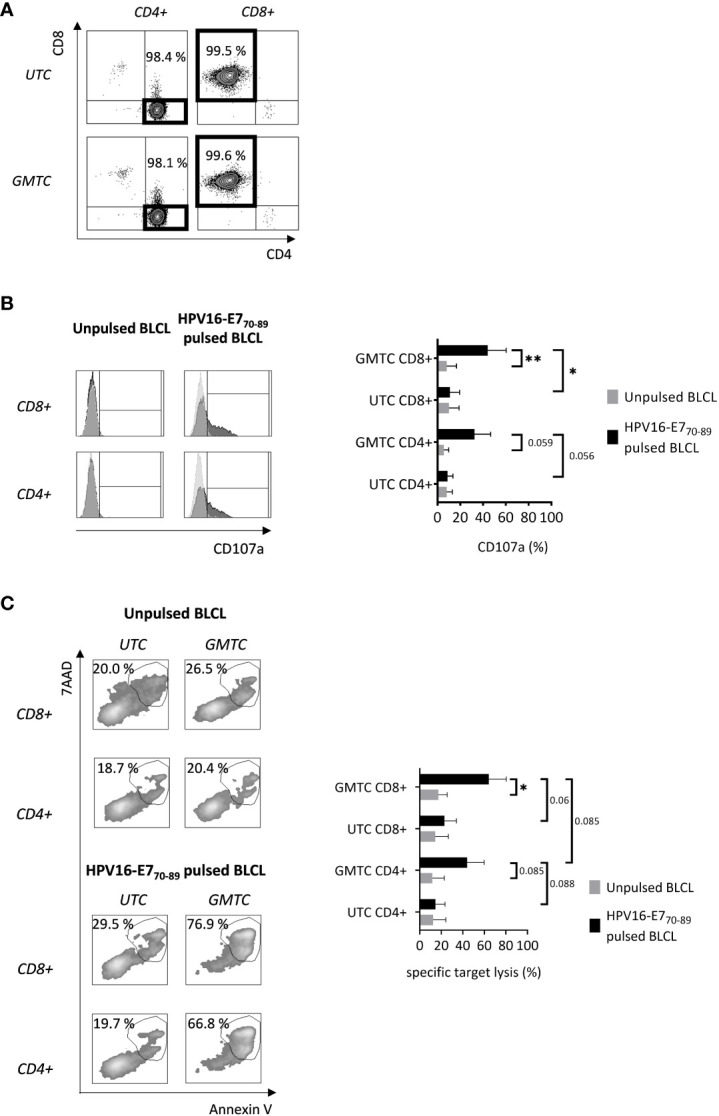
*In vitro* cytotoxic features of transduced CD8+ & CD4+ T cells. T cells are activated, cultured in the presence of IL-2, transduced (and selected) or not with HPV16-E7/HLA-DRB1*04-specific TCR-T, and sorted on the CD4 or CD8 co-receptors expression before being restimulated with HPV16-E7_70-89_-pulsed or not HLA-DRB1*04 BLCL. **(A)** CD4+ and CD8+ T cell sorting efficiency is evaluated through flow cytometry. Data are representative of 4 independent experiments. **(B)** Antigen-activated CD4+ and CD8+ T cell CD107a expression is evaluated through flow cytometry. Left: flow cytometry histograms representing CD107a expression of CD8+ or CD4+ T cells after restimulation; light and dark grey histograms corresponding to UTC and GMTC respectively; values obtained from one experiment, representative of 3. Right: bar graph representing CD107a expression of transduced or not CD8+ or CD4+ T cells after restimulation; mean+/-SD from 3 independent experiments; one-tailed paired t-test, *: p< 0.05 and **: p< 0.01. **(C)** CD4+ and CD8+ T cell-mediated target cell cytolysis is assessed through Annexin V/7AAD co-staining of target cells by flow cytometry. Left: flow cytometry plots representing Annexin V/7AAD expression of peptide-pulsed or not BLCL after co-culture with UTC or GMTC CD4+ or CD8+ T cells; values obtained from one experiment, representative of 3. Right: bar graph representing specific target lysis of transduced or not CD8+ or CD4+ T cells after restimulation; specific target lysis = [(% Annexin V+/7AAD+ _cocultured target_ - % Annexin V+/7AAD+ _alone target_)/(100 - % Annexin V+/7AAD+ _alone target_)] x 100; mean+/-SD from 3 independent experiments; one-tailed paired t-test, *: p< 0.05.

CD4+ and CD8+ were sorted from TCR-T and untransduced T cells after expansion in the presence of IL-2. Sorting efficiency is high enough [97.95% (mean)+/-1.14 (SD) and 98%+/-1.93 for CD4+ and CD8+ GMTC, respectively] to ensure that further observed results are attributed to sorted T cell subsets (**Figure 3A**). CD107a degranulation marker expression on sorted gene-modified CD8+ T cells (**Figure 3B**) is significantly increased after HPV16-E7_70-89_-pulsed BLCL co-culture when compared with either sorted unmodified CD8+ T cells or unpulsed BLCL co-culture experimental conditions (p< 0.05 and 0.01, respectively). A comparable upward trend is observed regarding CD107a expression by CD4+ T cells (**Figure 3B**), even if statistical significance at a 5% α risk is not fully achieved (p = 0.059 and 0.056 by comparing HPV16-E7_70-89_-pulsed BLCL co-cultured gene-modified CD4+ T cells with unpulsed BLCL and untransduced CD4+ T cells experimental conditions, respectively).

Cytotoxicity-related data perfectly mirror CD107a expression (**Figure 3C**). Indeed, HPV16-E7_70-89_ peptide-pulsed BLCL-specific target lysis is significantly higher after co-culture with transduced CD8+ T cells compared with unpulsed BLCL experimental conditions (p< 0.05). A similar strong trend is observed between transduced and untransduced CD8+ T cells (p= 0.06). Regarding CD4+ T cells, the differences observed after co-culture of either transduced T cells with peptide-pulsed or unpulsed target cells and transduced or untransduced T cells with peptide-pulsed target cells, are not statistically significant at a 5% α risk (p = 0.085 and 0.088, respectively). Nevertheless, the risk that these differences are only due to a random event is less than 9%. CD4+ TCR-T cells seem to exhibit less cytotoxic capacities than their CD8+ TCR-T counterparts, even if statistical significance is not fully achieved (p= 0.085).

Taken together, these results demonstrate that TCR-T II CD8+, and to a lesser extent CD4+, transduced T cells exhibit *in vitro* cytotoxic features toward cognate antigen-bearing target cells.

### Cytotoxic-associated CD4+ T cells phenotype evaluation of *ex vivo* activated and expanded T cells

**Figure 4:**
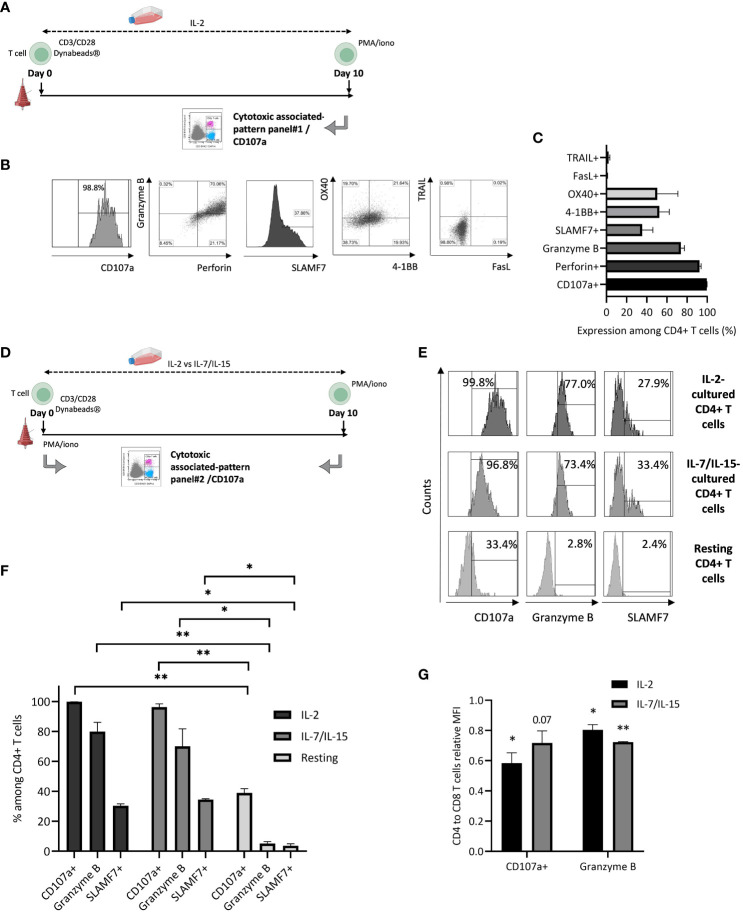
Cytotoxic phenotypic features of ex vivo cultured CD4+ T cells. **(A)** T cells are activated and cultured in the presence of IL-2 before PMA/iono restimulation and cytotoxicity-associated expression pattern evaluation (panel 1). Figure adapted from images created with BioRender.com. **(B)** Flow cytometry plots representing CD4+ T cell expression of CD107a, Granzyme B, Perforin, SLAMF7, OX40, 4-1BB, FasL, and TRAIL; data from one experiment, representative of 3. **(C)** Bar graph representing mean+/-SD from 3 independent experiments for all cytotoxicity-associated CD4+ T cell evaluated markers. **(D)** T cells are resting or activated and cultured in the presence of IL-2 or IL-7 and IL-15 before PMA/iono restimulation and cytotoxicity-associated expression pattern evaluation (panel 2). Figure adapted from images created with BioRender.com. **(E)** CD4+ T cell cytotoxicity-associated expression pattern: flow cytometry plots representing CD4+ T cell expression of CD107a, Granzyme B, SLAMF7 after restimulation or not; data from one experiment, representative of 3. **(F)** Bar graph representing mean+/-SD from 3 independent experiments for all cytotoxicity-associated CD4+ T cell evaluated markers, after restimulation; two-tailed paired t-test, *: p< 0.05 and **: p< 0.01. **(G)** CD4+ T cells degranulation marker expression intensity, relative to what is observed for their cytotoxic CD8+ T cell counterparts: T cells are either activated and cultured in the presence of IL-2 or IL-7/IL-15 before PMA/iono restimulation and degranulation marker expression intensity (Mean of Fluorescence Intensity or MFI) evaluation, through flow cytometry (panel 2). Bar graph represents mean+/-SD from 3 independent experiments. Pictured statistical analysis are made by comparing the mean of MFI observed for CD4+ and CD8+ T cells, for each culture condition; two-tailed paired t-test, *: p< 0.05 and **: p< 0.01.

*Ex vivo* culture conditions are likely to be involved in the development of CTX CD4+ T cells. We first hypothesized that IL-2 supplementation could be related to this phenomenon (**Figure 4A**). CD4+ T cell CD107a expression and cytotoxic profile were assessed after initial activation through CD3/CD28, *ex vivo* culture with 500 IU/mL IL-2 supplementation, and antigenic rechallenge mimicking PMA/ionomycin stimulation (**Figures 4B, C**). Stimulated CD4+ T cells express a high amount of CD107a, Granzyme B, and Perforin, and a significant amount of SLAMF7, OX40, and 4-1BB. A very low level of death receptors TRAIL and FasL co-expression is detected. This phenotype is consistent with a cytotoxic-associated CD4+ T cell phenotype.

We then evaluated the cytokine environment impact on CD4+ T cell cytotoxic features during *ex vivo* culture. CD107a, Granzyme B, and SLAMF7 expression were assessed for resting *versus* cultured T cells after initial activation through CD3/CD28 magnetic beads, *ex vivo* culture with either 500 IU/mL IL-2 or IL-7 350 IU/mL and IL-15 60 IU/mL supplementation and antigenic rechallenge mimicking PMA/ionomycin stimulation (**Figure 4D**). A similar expression pattern is observed regarding CD107a, Granzyme B and SLAMF7 expression after exposure to IL-2 compared to IL-7 and IL-15. Resting CD4+ T cell cytotoxic expression pattern is significantly different compared to the one of IL-2 or IL-7 and IL-15 exposed T cells (**Figures 4E, F**). CD107a and Granzyme B mean fluorescence intensities are lower for CTX CD4+ T cells compared to their classical cytotoxic CD8+ T cell counterparts. Similar results are obtained after both IL-2 (p< 0.05 regarding CD107a and Granzyme B) and IL-7/IL-15 (p= 0.07 and p< 0.01 regarding CD107a and Granzyme B, respectively) exposure (**Figure 4G**). This observation is consistent with previously observed data regarding cytotoxicity assessment (**Figure 3C**).

Altogether, these results demonstrate that our *ex vivo* T cell culture conditions are likely to induce CTX CD4+ T cells and that IL-2 is not the only responsible parameter for this phenomenon.

### *In vivo* cytotoxicity, infiltration and persistence capacity characterization of both CD8+ and CD4+ TCR-T II transduced T cells

We first validated the suitability of the SKMEL-28 cell line as an appropriate TCR-T target tumor cell for *in vivo* preclinical functionality studies. Indeed, membrane HLA-DR expression on SKMEL-28 cells was assessed (**Supplementary Figure S1A**). HPV-16 E7_70-89_-pulsed SKMEL-28 cells specifically induce TCR-T IFN-γ secretion, either from CD4+ (14.3%) or CD8+ (9.2%) cells, after an overnight co-culture (**Supplementary Figure S1B**). Both results suggest the ability of SKMEL-28 cells to present HPV-16 E7-derived peptide to TCR-T, in an MHC II restriction fashion. HPV16-E7 plasmid transfection efficiency and subsequent NeoR selection of SKMEL-28 cells were confirmed by Western blotting (**Supplementary Figure S1C**).

We then produced, as described above, a TCR-T batch including CD4+, CD8+, total GMTC as well as UTC from one healthy donor-derived T cells. This GMTC-specific lysis capacity was validated with CFSE-stained HPV16-E7_70-89_-pulsed HLA-DRB1*04 target cell line, either BLCL or SKMEL-28 cell line. Indeed, all GMTC subsets demonstrate a detectable cytotoxic capacity at an E:T ratio of 1:1. First, it is interesting to note that CD4+ GMTC are not as efficient in eliminating peptide-pulsed BLCL as their CD8+ counterparts or total GMTC. This result is consistent with Annexin V/7AAD staining results (**Figure 3C**). Second, all GMTC subsets demonstrate lower cytotoxic potential against peptide-pulsed SKMEL-28 cell line compared to BLCL. Moreover, CD8+ GMTC are less efficient to lyse peptide-pulsed SKMEL-28 cell line than their CD4+ counterpart and total GMTC population, in contrast to what occurs regarding BLCL (**Supplementary Figure S1D**). Overall, except for CD8+ GMTC which demonstrate the same low level of peptide-pulsed SKMEL-28 cell line-specific lysis at an E:T ratio of 1:1 and 1:5, all GMTC subsets show a decreased cytotoxicity against peptide-pulsed targets when diminishing the E:T ratio (**Supplementary Figure S1E**).

**Figure 5:**
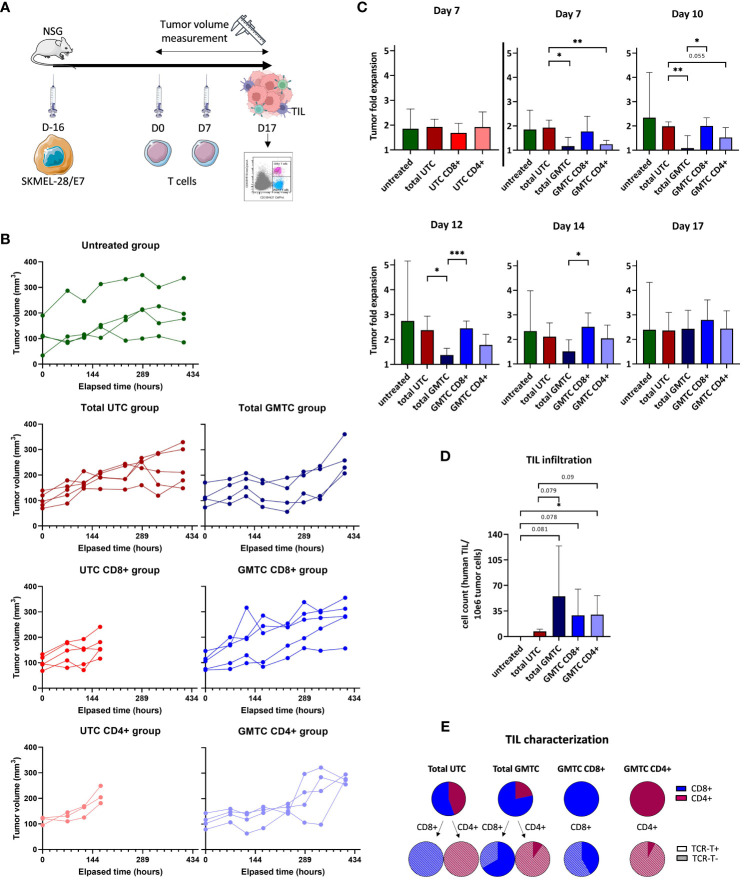
*in vivo* cytotoxic properties of CD4+ and CD8+ TCR-T cells evaluation **(A)** Experiment design. Figure adapted from images created with BioRender.com. and Servier Medical ART. **(B)** Tumor volume measurement for each mouse (green: untreated group, dark blue: total GMTC group, medium blue: CD8+ GMTC, light blue: CD4+ GMTC, dark red: total UTC, medium red: CD8+ UTC, light red: CD4+ UTC), at day 7, 10, 12, 14 and 17. **(C)** Tumor fold expansion for each mice group (mean+/-SD) (green: untreated group, dark blue: total GMTC group, medium blue: CD8+ GMTC, light blue: CD4+ GMTC, dark red: total UTC, medium red: CD8+ UTC, light red: CD4+ UTC), at day 7, 10, 12, 14, 17; two-tailed unpaired t-test, *: p< 0.05, **: p< 0.01 and ***: p< 0.001. **(D)** Number of human TIL/10^6^ tumor cell count for each mice group (mean+/-SD) (green: untreated group, dark blue: total GMTC group, medium blue: CD8+ GMTC, light blue: CD4+ GMTC, dark red: total UTC, medium red: CD8+ UTC, light red: CD4+ UTC); one-tailed unpaired t-test, *: p< 0.05. **(E)** TIL phenotypic characterization regarding CD4 (dark pink) or CD8 (blue) co-receptor and transgenic TCR expression or not (full and hatched part of pie charts).

When SKMEL-28/E7-derived tumor volume reached a mean of around 100 mm^3^ in NSG mice, all fractions of TCR-T and UTC cells were injected (**Figure 5A**). Tumor volume was calculated 3 times a week until sacrifice (**Figure 5B**) and tumor fold expansion ratio was calculated at Day (D)7, D10, D12, D14 and D17 post-treatment (**Figure 5C**). At D7, no effect on tumor growth control is observed regardless of the UTC subset. At D7 and D10, we demonstrate a tumor growth control mediated by CD4+ (p< 0.01 and p= 0.055, respectively for D7 and D10) and total GMTC (p< 0.01 and p< 0.05, respectively for D7 and D10), but not by CD8+ GMTC group, compared to total UTC control group. These data are consistent with the *in vitro* counterpart experiment shown in **Supplementary Figure S1B**. Tumor growth control mediated by CD4+ GMTC has the same amplitude but is less durable than the one mediated by the total GMTC. The second T-cell injection has no additional impact on tumor growth. From D14, GMTC-induced tumor growth control declines and is ultimately abolished at D17. Tumor infiltration by xenogeneic persistent T cells was assessed at D17, after sacrificing the mice. Few but detectable TIL are present in the tumor TCR-T-treated mice only (untreated vs total GMTC: p= 0.081; untreated vs CD8+ GMTC: p= 0.078; untreated vs CD4+ GMTC: p< 0.05; total UTC vs total GMTC: p= 0.079 and total UTC vs CD4+ GMTC: p= 0.09) (**Figure 5D**). Total GMTC and UTC TIL are composed of both CD4+ and CD8+ T cells. CD8+ and CD4+ GMTC TIL are only composed of CD8+ and CD4+ T cells, respectively. These data are consistent with what is expected according to effector subset quality controls shown in **Figure 3A**. Regarding the total UTC-injected mice group, we observe a trend to a fewer TIL infiltration and/or persistence in the tumor, when compared to the GMTC-injected mice. GMTC-derived TIL partially express TCR-T, with a trend to higher residual expression in CD8+ cells compared to CD4+ T cells (**Figure 5E**).

Taken together, these results emphasize the capacity of total GMTC and, to a lesser extent, of CD4+ GMTC, to delay the SKMEL-28/E7 growth in mice. Persistent infiltrating TCR-T, either CD8+ or CD4+, are detected in mice tumors and partially express the transgenic TCR.

## Discussion

*Ex vivo* retroviral transduction and TCR-T cell expansion allow for a cellular product constituted of both CD4+ and CD8+ T cells, transduced to a similar extent, consistent with previously published data by Schmueck et al. (30) and Dillard et al. (19). Consequently, these observations emphasized the systematic generation of a mismatch between T cell co-receptor and MHC transgenic TCR restriction. Regarding phenotypic characterization, we show here a lower GMTC expansion capacity during culture with IL-2, confirming data from Marton et al. (31). CD4/CD8 ratio is similar between GMTC and UTC, and is comparable to the one described for PBMC from healthy adults (32). Both subsets are present at levels considered as physiological in TCR-T and are prone to play a significant role in clinical settings. We do not demonstrate any differences between CD4+ and CD8+ T cells in terms of activation and low exhaustion profiles. Nevertheless, we observe a weak trend to higher T_PEX_ rate in CD4+ GMTC compared to UTC, which could be further investigated. In contrast to a CAR-T strategy where tonic signaling induced by antigen-independent triggering is prone to lead to T cell exhaustion, we did not expect an increase in either exhaustion or activation status of TCR-T (33).

In the present study on HPV16-E7 TCR-T, we confirm Dillard and colleagues’ *in vitro* data regarding cytotoxic CD8+ cell existence in a setting of MHC II-restricted TCR-T transduction, directed against the hTERT-derived peptide (19). We also demonstrate a strong trend of transgenic CD4+ T cells to exhibit similar cytotoxic capacities as CD8+ T cells. It has been previously demonstrated that the e*x vivo* T cell culture process favors Th1 CD4+ CTX T cell expansion rather than Treg expansion, possibly because of IL-2 bioavailability (9, 26). We confirm that culturing CD4+ T cells in the presence of IL-2 after initial activation is likely to induce strong CD107a expression and a cytotoxic phenotypic profile (10, 27) after a rechallenge-mimicking antigen exposure. We notice that these IL-2 exposed cell cytotoxic capacities seem to be mediated by the secretory Perforin/granzymes pathway, rather than by the death receptors pathway, as shown by the presence of Granzyme B/Perforin/CD107a and the absence of FasL and TRAIL in these CD4+ T cells. This associated cytotoxic phenotypic pattern is globally consistent with the one described at the mRNA level by Liang et al. (22). Thus, MART-1/HLA-A2 TCR-T CD4+ cells are able to transcribe gene coding for Granzyme B, Perforin, CD107a, SLAMF7, OX40, 4-1BB after antigen exposure; nevertheless, contrary to our present study, authors showed elements in accordance with an implication of both granule-dependent and independent killing pathways. Our data involving IL-2 or IL-7 and IL-15 supplemented cultured CD4+ T cells show similar results in terms of degranulation capacities and cytotoxic phenotypic profile. This element points out that IL-2 exposure is not the only way to favor the development of cytotoxic CD4+ T cells in *ex vivo* cultures. Nevertheless, we demonstrate a lower intensity of degranulation marker expression by CTX CD4+ T cells compared to their CTX CD8+ counterparts. These data are consistent with those obtained by Schober and colleagues (23), showing a faster decrease of Granzyme B secretion from CD4+ TCR-T compared to CD8+ TCR-T when reducing the E:T ratio. Then, in clinical settings, relatively less potent intrinsic cytotoxic capacities are likely to be expected from CTX CD4+ T cells. Nevertheless, any changes in the T cell *ex vivo* expansion step in a setting of ACT should be carefully evaluated regarding the CD4+ T cell cytotoxic status. Overall, we demonstrated that MHC II-restricted HPV16-E7_70-89_ GMTC CD4+ T cells display a cytolytic phenotype, thus confirming the studies of Kyte et al. (18), and that this phenotype is translated into cytotoxic function. This cytotoxicity potential is objectivated by *in vitro* target-specific killing as well as *in vivo* tumor control in mice. The *in vivo* experimentation, involving immunocompromised NSG mice and xenogeneic T cell graft, displays a valuable result regarding the differential capacity of CD4+, CD8+ and total TCR-T to control tumor growth. Indeed, a more durable response is obtained after total TCR-T injection. This result is consistent with the idea of a cooperation between CTX and helper T cell subsets to efficiently eradicate target cells. In the present model, CD4+ TCR-T are able to control tumor growth to a lesser extent, whereas CD8+ TCR-T are not. We hypothesize that CD4+ transgenic T cells, which acquired CTX properties during the *ex vivo* culture (even if their cytotoxic capacity is less intense than their CD8+ counterparts in terms of degranulation), have the capacity to supply both required cytotoxic and help features, contrary to CD8+ T cells which probably only exert cytotoxic function (34). Notably, we observe a discrepancy between *in vitro* and *in vivo* cytotoxicity assays regarding CD8+ TCR-T; these T cells are able to eliminate a cognate peptide-pulsed BLCL target cell line *in vitro*, but not the SKMEL-28 cell line *in vivo*, and to a lesser extent *in vitro*. We can assume that the lower HLA-DRB1*04 surface expression level displayed by the SKMEL-28 cell line compared to BLCL’s has an impact on TCR II/CD8+ mismatched T cells antigen recognition capacity, especially in a lack of help context. Another explanation can rely on the target cell line model, which can be sensitive to differential lytic pathways. Reverse settings, involving mismatched CD4+/TCR I TCR-T, have already been studied by Schober et al. (23) and Frankel et al. (24). In Schober’s study, CD4+ T cells display cytotoxic activity against cognate Ewing sarcoma target cells, both *in vitro* and *in vivo*, even if less efficiently compared to their matched-CD8+ TCR-T counterparts. Our study is in line with these results regarding the MHC/co-receptor match, even if the MHC context and the MHC/co-receptor mismatch are different and these features can influence the capacity of CD4+ and CD8+ TCR-T to eradicate tumor cells. Thus, the mismatch between MHC restriction and co-receptor expression could inherently limit for part the T cell functionalities. Interestingly, in Schober’s article, the *ex vivo* culture duration positively influences CD4+ T cell cytotoxic capacities. This issue could be addressed in our settings, even if positioned in a translational context. In Frankel’s work relying on tyrosinase expressing-melanoma models, unsorted, CD4+ and CD8+ TCR-T cells exert cytotoxic activity against cognate target tumor cell line, both *in vitro* and *in vivo*. All three subsets are able to induce a tumoral regression in mice at a similar level. In this setting, the mismatch between MHC restriction and co-receptor expression has no influence on tumor control, highlighting the difficulty of extrapolating previously described results in the TCR-T setting, obtained with a different study model. Furthermore, our study establishes that both CD8+ and CD4+ TCR-T cells are able to infiltrate the tumor and persist for part of them, even after tumor escape. TIL persistence could be reinforced for a potentially longer impact on the anti-tumoral immune response. Indeed, herein, TCR-T cells are expanded *ex vivo* in the presence of IL-2, which is known to induce more differentiated T cells. Replacing IL-2 with other cytokines such as IL-7 and IL-15 is likely to induce less differentiated T cells, retaining self-renewing potential in culture (31). Interestingly, we show that a fraction of TCR-T TIL does not express the transgenic TCR at the time of mice sacrifice. This lack of expression remains to be unraveled. Some hypothesis rely on a TCR-T expression loss during the *in vivo* experiment process or on a selective persistence of a minority ΔCD19-negative contaminating T cell subset.

Thereby, the existence and cytolytic activity of CTX CD4+ and CD8+ TCR-T, appropriately matched or not with the MHC expression, are assessed in this study and supply additional data to the existing literature. Miscommitment and/or MHC-mismatch seem to represent a weak limitation to T cell cytotoxicity in some models, without challenging TCR-T approaches and the different effector T cell subset role. At this stage, we can first expect simultaneous targeting of cytolytic and cognate helper fates against a given TAA through the transduction of TCR-T, either in an MHC I or MHC II context.

Certainly, MHC I is quite uniformly expressed by tumor cells, even if the loss of its expression is well known to be a cancer cell immune escape mechanism, while a great majority of tumor cells do not express MHC II, except in some hematologic malignancies or melanoma tumor cells. Thus, it could remain difficult for MHC II-restricted CD8+ or CD4+ T cells to be able to directly target a majority of cancer cells. Nevertheless, an indirect CD4+ T cells killing mechanism, implying IFN-γ secretion and tumoricidal macrophages, has already been reviewed and could represent the way for MHC II-restricted T cells to eliminate tumor cells, without any MHC I-restricted CD8+ T cells involvement (35). The same beneficial involvement could be expected for cytotoxic CD4+ T cells in the setting of antitumor CAR-T cells, because of the lack of MHC restriction involvement. A follow-up study of a CAR-T cell clinical trial (36) relates the *in vivo* persistence of cytotoxic characteristics-bearing CD4+ CAR-T cell clones more than 10 years after infusion. This tremendous persistence is associated with durable anti-tumor activity and patient long-term survival. Moreover, according to Yang et al. (37) in a mouse CAR-T cell setting, CTX CD4+ T cells remain insensitive to endogenous TCR engagement, contrary to CD8+ T cells which are prone to exhaustion after endogenous TCR triggering; these points are in favor of some expected CTX CD4+ T cell beneficial effect in this ACT context. Despite these CTX CD4+ TCR-T potential beneficial properties, the presence of these cells in ACT products should be considered with caution. Indeed, studies from Malek Abrahimians et al. (38) show the existence of CD4+ T cells able to acquire apoptosis-inducing properties on antigen-presenting cells and CD4+ MHC II expressing cells after cognate recognition of natural antigens and the opportunity of inhibiting these T cells to improve diabetes syndrome in a NOD mouse model. In this setting, CTX CD4+ T cells should be considered as a regulatory subset, potentially dysregulated in auto-immune diseases. However, these hypothetical deleterious effects, mediated by CTX CD4+ TCR-T within ACT products, should be mitigated by several observations. First, it has been known for a long time that dendritic cells are able to resist CTL lysis through an upregulation of Serpin Serine Protease Inhibitor 6 expression (39). Second, a recent study by Boulch et al. (40) showed that CAR-T cells-mediated-target-lysis is partly due to a cooperation with host immune cells.

So, we can secondly conclude that potential benefits associated with the presence of tumor-specific TCR-armed CTX CD4+ are now preclinically demonstrated. However, CTX CD4+ related deleterious effects could concomitantly occur. This issue should be taken into account when designing and developing a TCR-T-based ACT procedure, in the cancer treatment setting. Today, CD4+ and CD8+ TCR-T bulk injection is the more common approach reported in the clinical literature and relies on a global evaluation of specific anti-tumoral activity. According to present knowledge, the ideal composition of TCR-T in terms of CTX CD4+ and CD8+ T cell presence and ratio remains questionable and is likely to differ according to either the MHC context or evaluated tumor model, regarding efficiency indicators. Regardless of the engineered T cell-mediated approach, interactions between tumor-reactive modified T cells and other host immune cells related to toxicity-mediated impacts need to be carefully evaluated in clinical settings.

## Data availability statement

The raw data supporting the conclusions of this article will be made available by the authors, without undue reservation.

## Ethics statement

The studies involving humans were approved by CODECOH (collection agreement number AC-2020-4129). The studies were conducted in accordance with the local legislation and institutional requirements. The participants provided their written informed consent to participate in this study. The animal study was approved by Ministry of Agriculture (number 2021-004-OA12PR). The study was conducted in accordance with the local legislation and institutional requirements.

## Author contributions

PM-L designed the study, performed the experimental work, analyzed the data and wrote the manuscript. AK and CM reviewed and corrected the manuscript. OA, YG were involved in the management of the research work. JG supervised the work. All authors contributed to the article and approved the submitted version.

## Supplementary material

Supplementary Figure 1
*In vivo* cytotoxicity assay-designed cellular tools validation. **(A-C)** Characterization of SKMEL-28 as a target cell line. **(A)** HLA-DR expression: comparison of SKMEL-28 cell line and BLCL. The light grey peak and dark grey peak represent unstained and HLA-DR-stained cell line, respectively. Ratio of fluorescence intensity between the two peaks (RFI) is plotted on each graph. **(B)** Untransduced and transduced T cells are co-cultured with 1:1 SKMEL-28 cells, pulsed or not with the HPV16-E7_70-89_ peptide, then evaluated for IFN-γ secretion (CD4+ and CD8+ T cell subsets). **(C)** HPV16-E7 validation expression of transfected SKMEL-28 cells by Western Blotting. **(D, F)** Characterization of effector GMTC subsets batch *in vitro* cytotoxicity. **(D)** Representation of residual alive CFSE labelled-target cells [pulsed or not BLCL (black and medium grey bars) and pulsed or not SKMEL-28 cell line (dark and light grey bars)] after co-culture with effector T cells (total, CD8+ and CD4+ GMTC) at an effector:target ratio of 1:1. Specific target lysis = (Number of alive targets/Number of seeded targets) x 100. **(E)** Representation of residual alive CFSE labelled-target cells [pulsed BLCL (left) or SKMEL-28 cell line (right)] after coculture with effector T cells [total (circle), CD8+ (square) and CD4+ (triangle) GMTC] at an effector:target ratio of 1:1 and 1:5. Specific target lysis = (Number of alive targets/Number of seeded targets) x 100.
