# Supplementary figures and images for: Characterization of atypical T cells generated during ex vivo expansion process for T cell-based adoptive immunotherapy
Source: Front Immunol. 2024 Mar 13;15:1202017. doi: 10.3389/fimmu.2024.1202017 (PMC10965654; doi:10.3389/fimmu.2024.1202017)

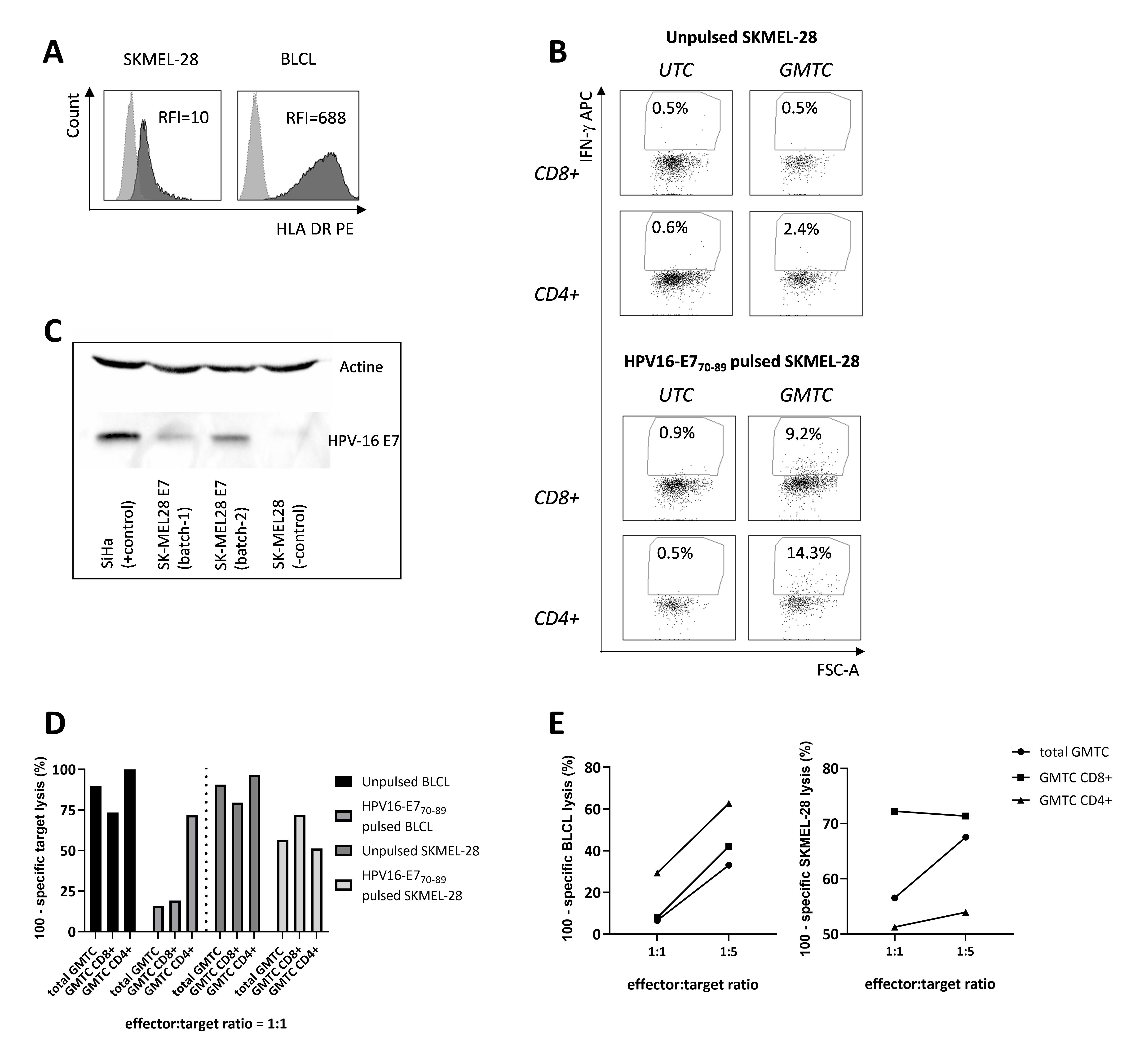

Supplement: Supplementary Figure 1 — In vivo cytotoxicity assay-designed cellular tools validation. (A-C) Characterization of SKMEL-28 as a target cell line. (A) HLA-DR expression: comparison of SKMEL-28 cell line and BLCL. The light grey peak and dark grey peak represent unstained and HLA-DR-stained cell line, respectively. Ratio of fluorescence intensity between the two peaks (RFI) is plotted on each graph. (B) Untransduced and transduced T cells are co-cultured with 1:1 SKMEL-28 cells, pulsed or not with the HPV16-E770-89 peptide, then evaluated for IFN-γ secretion (CD4+ and CD8+ T cell subsets). (C) HPV16-E7 validation expression of transfected SKMEL-28 cells by Western Blotting. (D, F) Characterization of effector GMTC subsets batch in vitro cytotoxicity. (D) Representation of residual alive CFSE labelled-target cells [pulsed or not BLCL (black and medium grey bars) and pulsed or not SKMEL-28 cell line (dark and light grey bars)] after co-culture with effector T cells (total, CD8+ and CD4+ GMTC) at an effector:target ratio of 1:1. Specific target lysis = (Number of alive targets/Number of seeded targets) x 100. (E) Representation of residual alive CFSE labelled-target cells [pulsed BLCL (left) or SKMEL-28 cell line (right)] after coculture with effector T cells [total (circle), CD8+ (square) and CD4+ (triangle) GMTC] at an effector:target ratio of 1:1 and 1:5. Specific target lysis = (Number of alive targets/Number of seeded targets) x 100. [file Image_1.jpeg]
